# Rat Glioma Cell-Based Functional Characterization of Anti-Stress and Protein Deaggregation Activities in the Marine Carotenoids, Astaxanthin and Fucoxanthin

**DOI:** 10.3390/md17030189

**Published:** 2019-03-23

**Authors:** Sajal Afzal, Sukant Garg, Yoshiyuki Ishida, Keiji Terao, Sunil C. Kaul, Renu Wadhwa

**Affiliations:** 1 DAILAB, DBT-AIST International Center for Translational & Environmental Research (DAICENTER), National Institute of Advanced Industrial Science & Technology (AIST), Tsukuba 305-8565, Japan; sajal.afzal@aist.go.jp (S.A.); sukantgarg@gmail.com (S.G.); s-kaul@aist.go.jp (S.C.K.); 2 School of Integrative and Global Majors, University of Tsukuba, Tsukuba 305-8577, Japan; 3 CycloChem Co., Ltd., 7-4-5 Minatojima-minamimachi, Chuo-ku, Kobe 650-0047, Japan; yoshiyuki.ishida@cyclochem.com (Y.I.); keiji.terao@cyclochem.com (K.T.)

**Keywords:** marine carotenoid, ultraviolet radiation, DNA damage, protein misfolding, protein aggregation, glial differentiation, protection

## Abstract

Stress, protein aggregation, and loss of functional properties of cells have been shown to contribute to several deleterious pathologies including cancer and neurodegeneration. The incidence of these pathologies has also been shown to increase with age and are often presented as evidence to the cumulative effect of stress and protein aggregation. Prevention or delay of onset of these diseases may prove to be unprecedentedly beneficial. In this study, we explored the anti-stress and differentiation-inducing potential of two marine bioactive carotenoids (astaxanthin and fucoxanthin) using rat glioma cells as a model. We found that the low (nontoxic) doses of both protected cells against UV-induced DNA damage, heavy metal, and heat-induced protein misfolding and aggregation of proteins. Their long-term treatment in glioma cells caused the induction of physiological differentiation into astrocytes. These phenotypes were supported by upregulation of proteins that regulate cell proliferation, DNA damage repair mechanism, and glial differentiation, suggesting their potential for prevention and treatment of stress, protein aggregation, and age-related pathologies.

## 1. Introduction

Stress has been largely defined as a causative factor for a variety of diseases and premature aging as marked by the early occurrence of age-related decline in body functions [1]. On these premises, there is a continuous search and enrollment of natural compounds with anti-stress potential for extending quality of life in normal and disease scenarios. Cell-based functional assays provide a useful model system to identify such physiologically relevant compounds [2]. Oxidative and UV-induced stresses are some of the most common stresses that cause molecular damage both at the DNA and protein level [3], and are often linked to the lethal pathologies including cancer and brain dysfunctions [1,4]. Prevention against stress-induced molecular damage may be useful for disease prevention and/or treatment. Furthermore, assignment of functional properties (such as differentiation to perform specific functions) to cancer cells, that most frequently lose their functional characteristics and de-differentiate to divide indefinitely, could prove to be a highly useful nontoxic way of treating cancer [5]. In view of this, we chose glioma that possesses the capability to get differentiated into glia/astrocytes. The latter is composed abundantly in intracranial soft tissue, and functions as a major supporting tissue serving anchorage, nourishment, oxygenation, insulation, and excretion to the neurons and various other brain functions. Furthermore, glioma is one of the lethal cancers with a poor prognosis and survival rate [6]. Whereas glial differentiation is well marked by upregulation of GFAP (glial fibrillary acidic protein), PSD95 (postsynaptic density protein 95), MAP2 (microtubule-associated protein 2), and GAP43 (growth-associated protein 43) proteins, glioma often shows either downregulation or lack of these proteins [7,8,9,10]. Natural compounds that could induce the upregulation of these proteins and induce glial differentiation have attracted a lot of attention in research laboratories due to their easy availability, relatively lower toxicity index, and economic aspects [11,12].

Some of the relatively unexplored and reasonably newer varieties of natural molecules are those produced in marine organisms. Marine bacteria, fungi, algae, and archaea are healthy and rich sources of certain carotenoids. The latter are either carotenes (pure hydrocarbons) or xanthophylls (oxygenated hydrocarbons) that contribute to distinctive pigmentation of the marine organisms [13]. Astaxanthin and fucoxanthin, as shown in Supplementary Figure S1, are two xanthophylls that are known for their antioxidant and cytoprotective functions [14], and therefore have gained attention in the health, food, and cosmetic industries. Astaxanthin, commonly found in *Haematococcus pluvialis* and *Phaffia rhodozyma*, has been shown to possess potent antioxidant and anti-inflammatory activities [14,15,16]. It showed reactive oxygen species (ROS) and free radical scavenging properties as a quencher of singlet reactive oxygen, nitrogen, as well as single and two electron oxidants. Astaxanthin has also been shown to offer protection against Alzheimer’s disease [17], brain injury [18], cardiac injury [19], contrast-induced nephropathy [20], and muscle atrophy [21]. It is not synthesized by the human body and needs to be obtained exogenously. Since its over-consumption has not been known to cause any toxicity, it has been approved and widely marketed by the United States Food and Drug Administration as a dietary supplement [22,23]. Fucoxanthin, on the other hand, characterized by its distinctively strong orange tint and commonly found in *Phaeophyceae* and *Bacillariophyta*, has also been shown to possess similar therapeutic properties [24,25]. However, it was found to be unstable and easily degraded by light, heat, and oxygen [26]. Nevertheless, it has been shown to inhibit proliferation in gastric cancer cells [27], induce autophagy followed by apoptosis in gastric cancer cells at a significantly higher dose [28], and possess potent anticancer effects against glioma cells [29] at a relatively wider range of doses. Fucoxanthin, at higher doses, causes apoptosis and inhibition in cell proliferation by suppression of the PI3K/Akt/mTOR survival pathway and inhibition of their metastatic profiles at relatively lower doses. Similarly, it also protected neurons against ischemic/reperfusion injury [30] and liver cancer cells against tributyltin-induced oxidative injury [31] through the activation of anti-stress heme oxygenase signaling. It has also been shown to protect mice against dextran sulfate sodium-induced colitis [32] and UVB-induced skin erythema [33]. Interestingly, it also extended the overall lifespan in a Drosophila model by triggering distinct genetic modifications [34].

Considering this information, we investigated anti-stress potentials of astaxanthin and fucoxanthin using cell-based biochemical assays. We found that the nontoxic doses of astaxanthin and fucoxanthin protected these cells against UV stress. The treated cells showed astrocytic differentiation characteristics. The reversal of the DNA damage, protein misfolding, and protein aggregation stresses was also observed in cells treated with astaxanthin and fucoxanthin suggesting their potential in the treatment of old-age related pathologies that involve cumulative DNA and protein damage.

## 2. Results

### 2.1. Toxicity Profile of Astaxanthin and Fucoxanthin in Rat Glioma

**Figure 1:**
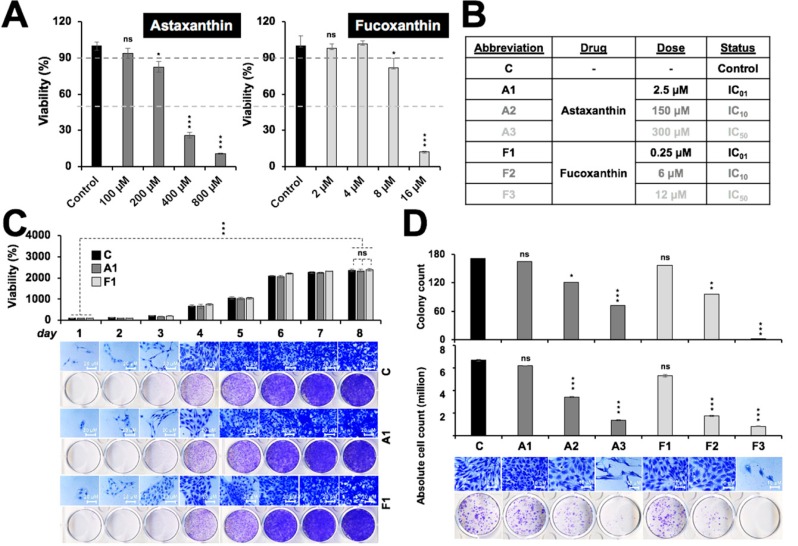
In vitro toxicity profiling of the marine carotenoids. (**A**) Dose-dependent cytotoxicity of astaxanthin and fucoxanthin in C6 cells as determined by cell viability assays. (**B**) IC_01_, IC_10_, and IC_50_ doses of astaxanthin and fucoxanthin as determined by dose-dependent cytotoxicity titration curves. (**C**) Long-term cell proliferation analysis with the safe doses of astaxanthin (A1) and fucoxanthin (F1) showing no effect on proliferation or cell morphology of C6 cells. (**D**) Quantitative cell viability (QCV) assay, depicting insignificant inhibition of clonogenicity or intracellular stress at the safe dose of both astaxanthin and fucoxanthin. Statistical significance was calculated by an unpaired *t*-test of GraphPad^®^ software (2018) using mean, SD, and N from three independent experiments, and shown as * *p* < 0.05, ** *p* < 0.01, *** *p* < 0.001, ns = not significant.

We first examined the toxicity of astaxanthin (Asta) and fucoxanthin (Fuco) on rat glioma cells. As shown in Figure 1A, astaxanthin caused toxicity at doses >200 µM. On the other hand, fucoxanthin treated cells showed toxicity at doses 8 µM and above. Approximate IC_50_ and IC_10_ and IC_01_ doses, as derived from three independent experiments, are tabulated in Figure 1B. Based on these data, 2.5 µM Asta (A1) and 0.25 µM Fuco (F1) were chosen as the nontoxic doses. Both A1 and F1 treated cells showed no significant difference, with respect to control cells, both on short- and long-term viability, clonogenicity as well as cell morphology as observed by a quantitative cell viability assay [2] and cell proliferation assay, as shown in Figure 1C,D. Therefore, these nontoxic doses were used for further experiments.

### 2.2. Nontoxic Doses of Astaxanthin and Fucoxanthin Protected Cells against DNA Damage Stress

**Figure 2:**
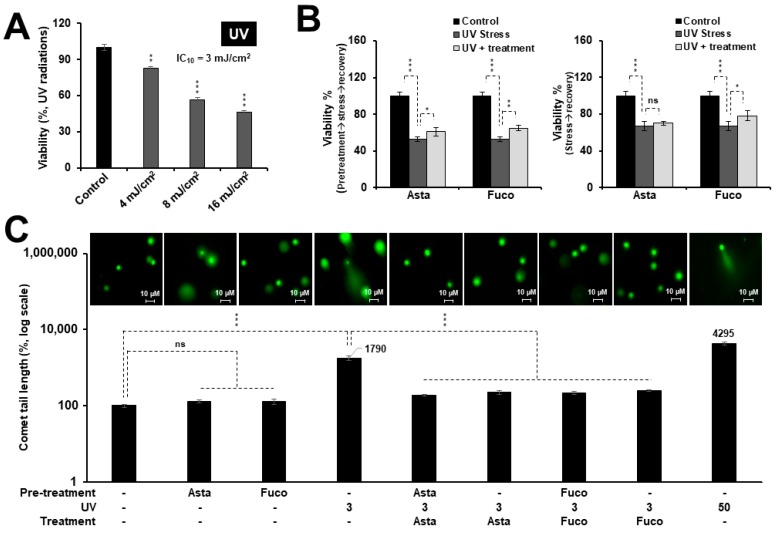
Low nontoxic doses of Asta/Fuco protected C6 cells against UV-induced DNA damage. (**A**) Effect of UV radiation on the viability of C6 cells. (**B**) UV-responsive cell viability assay showing, small but significant, increase in viability of treated cells; cells pretreated with Asta/Fuco showed stronger effect (left) as compared to the ones treated only after the UV exposure (right). (**C**) Neutral comet assay showing protection against UV-induced DNA damage in cells treated with Asta/Fuco. Statistical significance was calculated by an unpaired *t*-test of GraphPad^®^ software (2018) using mean, SD, and N from at least three independent experiments, and shown as * *p* < 0.05, ** *p* < 0.01, *** *p* < 0.001, ns = not significant.

**Figure 3:**
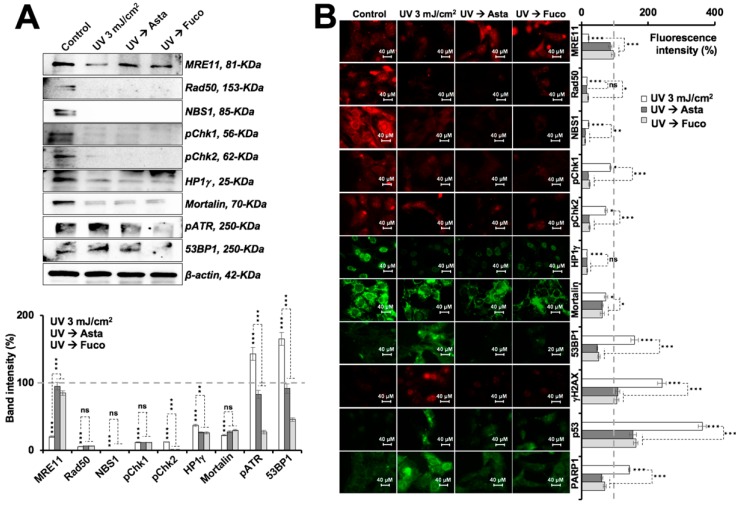
Effect of low nontoxic doses of Asta/Fuco on proteins involved in UV-induced DNA damage signaling. Immunoblotting (**A**) and immunostaining (**B**) of MRN complex and DNA damage response proteins in control and treated cells. Statistical significance was calculated by an unpaired *t*-test of GraphPad^®^ software (2018) using mean, SD, and N from at least three independent experiments, and shown as * *p* < 0.05, ** *p* < 0.01, *** *p* < 0.001, ns = not significant.

C6 cells were subjected to UV and their IC_10–30_ doses were determined by several independent experiments, as shown in Figure 2A. Next, UV (IC_10_) treated cells were further treated with Asta or Fuco. As shown in Figure 2B, 5 mJ/cm^2^ of UV radiation caused about 30–50% loss in cell viability over a period of 48 h. Notably, although to a small extent, both Asta and Fuco treatment caused significant recovery with pretreatment, as shown in Figure 2B (left panel), or without pretreatment, as shown in Figure 2B (right panel). UV radiation induces double-strand DNA damage and mutagenesis [35]. A comet assay—a standard method to analyze DNA damage—was performed to check the extent of UV-induced DNA damage and its potential protection by Asta and Fuco. As shown in Figure 2C, 3 mJ/cm^2^ of UV radiation caused considerable (about 18-fold) DNA damage in C6 cells that was significantly limited by both Asta and Fuco supplementation before or after the exposure. In order to address the mechanism of such protection, we next examined the expression of proteins related to proliferation and DNA damage in control and treated cells. Cells stressed with UV and recovered in control/Asta/Fuco supplemented medium were harvested for immunoblotting and immunostaining for various proteins using specific antibodies. As shown in Figure 3A,B, exposure to 3 mJ/cm^2^ UV radiation caused downregulation of MRN complex, Chk1/2 activation, HP1γ, and mortalin, and upregulation of DNA damage markers 53BP1 and phosphorylated ATR. Cells that were recovered in Asta or Fuco supplemented medium showed significant recovery in MRE11 expression. Furthermore, increase in DNA damage markers (pATR and 53BP1) was abrogated. An immunofluorescence assay confirmed these data and also demonstrated an increase in DNA damage signifying proteins γH2AX, p53, and its downstream PARP1 in cells exposed to UV; the increase was attenuated by Asta or Fuco treatment. Rad50, NBS1, Chk1, Chk2, HP1γ, and mortalin did not show significant changes.

### 2.3. Nontoxic Doses of Astaxanthin and Fucoxanthin Prevented Protein Aggregation and Protein Misfolding

**Figure 4:**
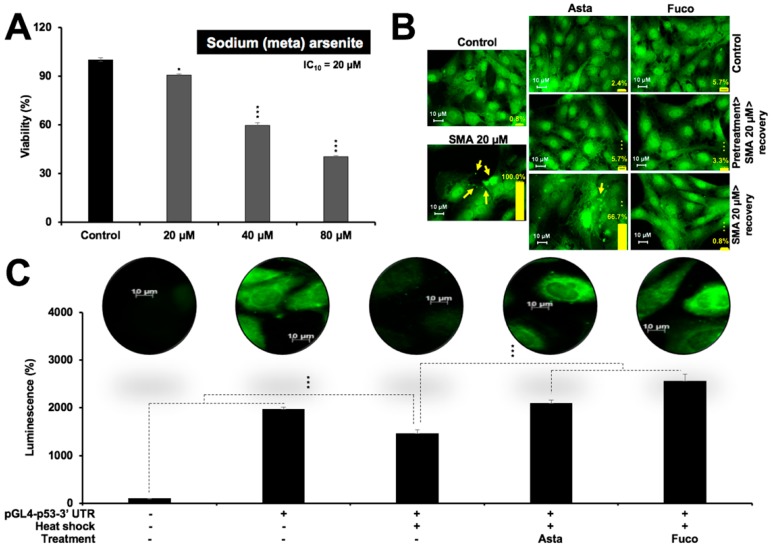
Effect of low doses of Asta/Fuco on heavy metal and heat-shock-induced protein aggregation. (**A**) Effect of sodium (meta)arsenite (SMA) on viability of C6 cells. (**B**) Protein aggregation and deaggregation assay, showing the GFP aggregation in SMA-treated cells and deaggregation when subsequently treated with Asta/Fuco. Cells pretreated with Asta and Fuco showed strong deaggregation. (**C**) Heat-shock responsive luciferase reporter (pGL4-p53-3′ UTR) in control, heat-shocked (control and Asta/Fuco treated) cells showing significant protection against the heat-shock stress in the latter. Statistical significance was calculated by an unpaired *t*-test of GraphPad^®^ software (2018) using mean, SD, and N from three independent experiments, and shown as * *p* < 0.05, *** *p* < 0.001.

DNA damage and protein aggregation are the key hallmarks of several diseases including several old age-related brain pathologies. We next examined the effect of Asta and Fuco on protein aggregation using metal-induced protein aggregation as the model [36]. C6 cells were treated with a nontoxic (IC_10_) dose of sodium (meta)arsenite, as shown in Figure 4A. In order to record the protein aggregation visually, cells were tagged with GFP. As shown in Figure 4B, treated cells showed microscopically appreciable aggregation of GFP. Of note, pretreatment of cells with Asta and Fuco showed clear abolishment of aggregated GFP whereas recovery of cells in the presence of Fuco was equally effective. The aggregates of GFP seen in the cytoplasm of the stressed cells were seen to disappear (deaggregate) when they were treated with Asta or Fuco. Aggregation of the proteins is a common phenomenon found in the pathogenesis of various chronic diseases. We next confirmed such effect of Asta and Fuco using heat-shock-induced protein misfolding of luciferase assays. Cells were transfected with a luciferase-expressing plasmid. Misfolding of luciferase was induced by heat-shock treatment [37]. The effect of Asta and Fuco on protein misfolding was determined quantitatively by luciferase assays. Cells were also immunostained with anti-luciferase antibody to record the expression level and distribution of luciferase. As shown in Figure 4C, heat shock inhibited the expression of luciferase that was significantly reversed with the treatment of Asta and Fuco.

### 2.4. Nontoxic Doses of Astaxanthin and Fucoxanthin Induced Differentiation in Glioma Cells

**Figure 5:**
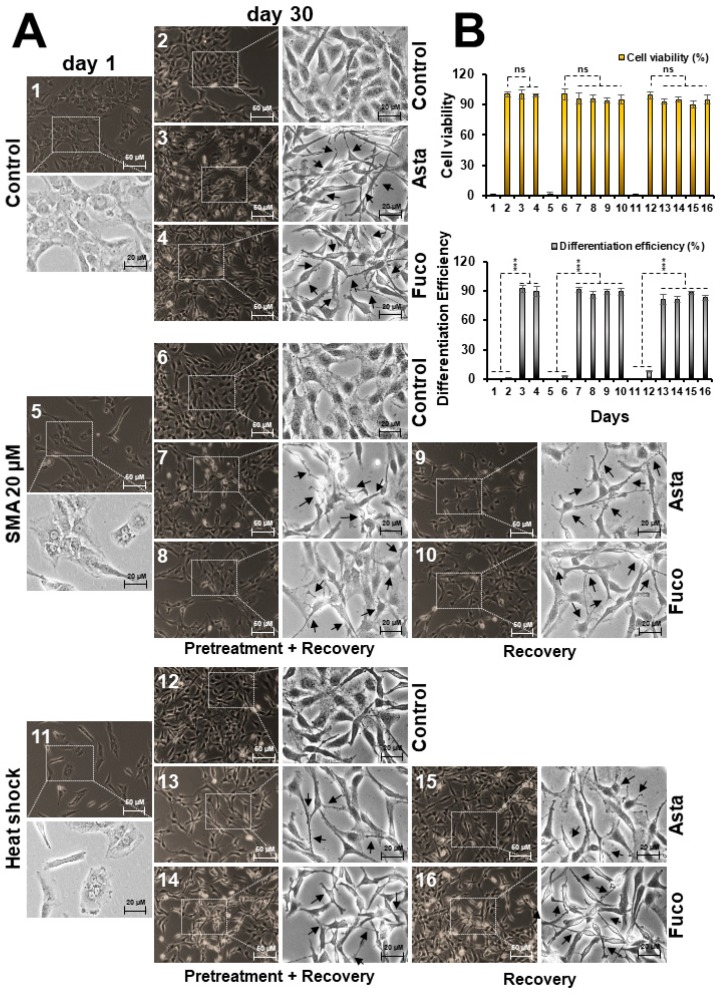
Low doses of astaxanthin and fucoxanthin caused glial cell differentiation (**A**) and its quantification (**B**) for cell viability and differentiation. Statistical significance was calculated by an unpaired *t*-test of GraphPad^®^ software (2018) using mean, SD, and N from three independent experiments, and shown as *** *p* < 0.001, ns = not significant.

**Figure 6:**
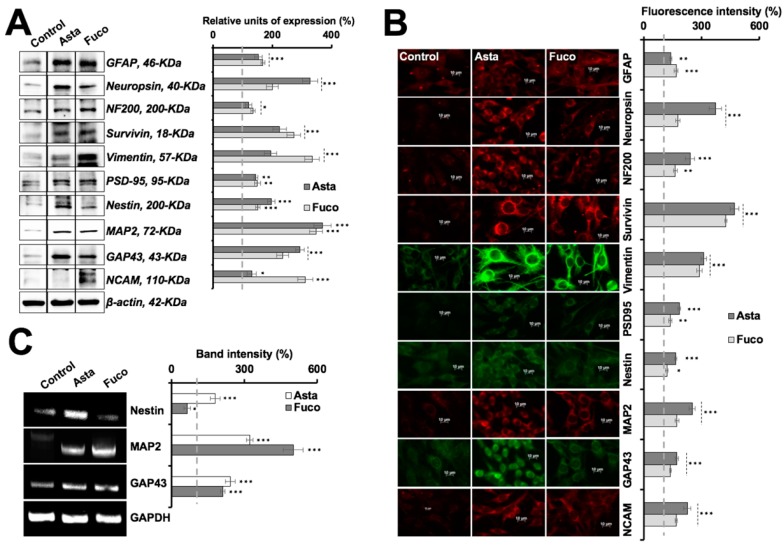
Expression analysis of key regulators of glial differentiation in control, Asta- and Fuco-treated cells. (**A**,**B**) Immunoblotting and immunostaining of cells, showing upregulation differentiation-associated proteins in treated cells. (**C**) RT-PCR analysis, showing that MAP2 and GAP43 proteins are transcriptionally upregulated in treated cells; nestin showed upregulation in Fuco-treated cells only. Statistical significance was calculated by an unpaired *t*-test of GraphPad^®^ software (2018) using mean, SD, and N from at least three independent experiments, and shown as * *p* < 0.05, ** *p* < 0.01, *** *p* < 0.001.

Induction of differentiation in brain cancer cells is an important aspect of the therapeutic rationales. We, therefore, investigated the differentiation-inducing potential of Asta and Fuco. As shown in Figure 5A, treatment of C6 cells with Asta and Fuco (the IC_01_ doses for 30 days) caused the emergence of cells that appeared like differentiated astrocytes (radiating dendrites, soma hypertrophy, process thickening, and axonal reconnections) [38,39]. Of note, cells stressed with either exposure to sodium (meta)arsenite (SMA) or heat shock also showed similar differentiation upon subsequent culture in Asta/Fuco supplemented medium, as shown in Figure 5A,B. There was no significant loss of cell viability observed, as shown in Figure 5B. In order to confirm the astrocytic differentiation, at the molecular level, we examined the expression of protein markers specific to differentiation. As shown in Figure 6A,B, expressions of GFAP, neuropsin, NF200, survivin, vimentin, PSD-95, nestin, MAP2, GAP43, and NCAM proteins were significantly upregulated. RT-PCR analyses revealed that MAP2, GAP43, and nestin were upregulated at the transcriptional level in Asta-treated cells, as shown in Figure 6C. Fuco-treated cells, on the other hand, showed transcriptional upregulation of MAP2 and GAP43; nestin showed a slight decrease. These data strongly suggest that astaxanthin and fucoxanthin have significant glial cell differentiating capacity and may work through common, as well as different, signaling pathways.

## 3. Discussion

Carotenoids from several marine organisms such as microalgae and diatoms are considered and have previously been shown to possess excellent anti-oxidant properties [14,16,24]. Marine carotenoids sources confer with numerous advantages viz., low cost, easy availability, high chemotherapeutic and nutritional index, and low contamination due to their halophilic habitats [40]. Several carotenoids have been isolated and utilized in the pharmaceutical, nutraceutical, and cosmeceutical industries. Understanding their molecular mechanism of action is vital in order to comprehend and rehearse their sustainable and organized use.

Astaxanthin and fucoxanthin are two marine carotene xanthophylls that have been shown to possess powerful anti-oxidant activity owed to their unique structure (epoxide groups, allenic bonds, conjugated double bonds, and conjugated carbonyl groups in the polyene chain) and high potential to quench singlet oxygen and hence act as free radical scavengers [41]. Based on this, they have been predicted to possess preventive and therapeutic potential for oxidative stress-mediated diseases [14,40]. The latter are marked by increased production and accumulation of reactive oxygen species (ROS) that possess the ability to damage cells by oxidizing DNA, proteins, and lipids and worse, mutate them. One of the most commonly explained mechanisms of action of stress to disease transformation is via protein misfolding causing structural changes in the cytoskeleton [37]. It has been firmly established that while the acute exposure to oxidative and DNA damaging ultraviolet radiation causes the production of ROS and double-stranded breaks in the DNA. Anomalies in DNA damage repair mechanisms often cause mutations and lead to the development of cancer, early aging, and several degenerative diseases. Chronic exposure to these stresses leads to continual protein misfolding and aggregation [42,43]. These aggregates contribute to age-related degenerative pathologies to a large extent [1,44,45,46]. Most recently, cancer has been defined as one of the old-age related pathologies, partly due to extended human life-span and increased level of chemical and environmental stresses [47]. Cancer diagnostics and therapeutics have made remarkable progress in the last two to three decades [48]. A large number of anticancer drugs have traveled from the laboratory to clinics. However, most of the drugs are synthetic and are associated with severe adverse effects and drug resistance. To overcome this problem, the discovery and development of several alternate means of anti-stress therapies are on the rise. One of the recently introduced targeted therapies applicable to brain-related cancers is the induction of cancer cell differentiation [5]. Owing to the high prevalence of brain cancers, the high rate of carcinogenic de-differentiation in brain tissues, and their failure to regenerate after chemotherapy-mediated cytotoxicity, cancer cell differentiation may be considered one of the most rewarding antitumor remedies.

Comparable to the conventional remedies, astaxanthin and fucoxanthin showed both anti-stress and differentiation-inducing potential in rat glioma cells. Of note, stress (radiation, temperature, and heavy metal poisoning)-induced molecular changes were reversed in some, if not all, when cells were treated with astaxanthin and fucoxanthin. Ultraviolet radiation has been shown to cause indirect damage to the DNA via absorption of photons by the non-DNA chromophores and a resultant ROS formation that oxidize DNA bases causing mutations [49], triggering the DNA damage response signaling pathway orchestrated by the key regulator ATR kinase recruiting proteins such as the MRN complex, 53BP1, and BRCA, and functional repression of proliferation via the expression of HP1γ [50,51,52]. We found that the culture of UV-stressed C6 cells in astaxanthin- or fucoxanthin-supplemented medium led to the suppression of the DNA damage markers pATR and 53BP1, and partial reactivation of the MRN complex, as shown in Figure 3. The mechanism of action of protection against UV radiation by astaxanthin and fucoxanthin in glioma cells could be attributed to their ability to reactivate the MRN complex and its downstream factor 53BP1, leading to the reciprocal phosphorylation of ATR, thereby facilitating the process of DNA damage repair inhibition, as shown in Figure 2B,C. On the other hand, HP1γ and mortalin (chromatin modulating and stress chaperone proteins, respectively) showed a decrease in UV-stressed control as well as treated cells suggesting that astaxanthin and fucoxanthin may work predominantly through protecting the cells against DNA damage [53]. The latter has been implicated in chronic protein aggregating degenerative diseases [54,55,56]. Heavy metals have been known to interfere with protein homeostasis and stabilization, via formation of toxic P-bodies (or stress granules) that aggregate and co-sediment with several heat-shock chaperones leading to the protein misfolding [36,57]. Proteinopathies resulting from long-term protein misfolding and/or failure of the cells to eliminate misfolded proteins have been known to be one of the major causes of old-age related pathologies [58]. A number of neurodegenerative diseases are marked by the hallmark events of protein misfolding, and their aggregation and accumulation that further cause a loss of synaptic connections and cellular dysfunction in brain tissue. Both astaxanthin and fucoxanthin caused deaggregation of the sodium (meta)arsenite-induced aggregated GFP protein, as shown in Figure 4A,B, and inhibited heat-shock-induced folding of luciferase protein, as shown in Figure 4C. Our results suggest the plausible ability of astaxanthin and fucoxanthin to protect the cells against heavy metal or heat-induced stress including protein aggregation and molecular damage, thereby useful against protein aggregation/misfolding mediated pathologies.

Astaxanthin and fucoxanthin both strongly caused differentiation in C6 cells into the respective functional phenotypes (as supported by molecular changes), signifying that they could serve as important and attractive differentiation-based therapeutic agents. Cells treated with astaxanthin and fucoxanthin showed characteristics of astrocytes including radiating dendrites, flattened morphology, soma hypertrophy, and cell-to-cell connections with synaptic junctions [38,39]. Molecular analysis indeed revealed upregulation of proteins involved in functional neurogenesis. The differentiated cells demonstrated a distinct upregulation pattern in the expression of MAP2 (morphogenesis regulator and organelle trafficker) and PSD-95 (post-synaptic targeting scaffolder) [59], GAP43 (key protein in growth cone formation, neurite outgrowth, and development of functional cerebral cortex) [60], GFAP and vimentin (shape and motility modulators) and nestin (neural cell division and migration regulator) [61], neuropsin (functional photoreceptor) [62], NCAM (responsible for neuronal attachment, extension, and cell-to-cell interaction) [63] and survivin (cell survival and proliferation enhancer) [62] proteins. These signature proteins may also endorse the reconstruction of glioma cells into mature neuronal and Schwann cell phenotypes, as shown in Supplementary Figure S2. These data suggest that the marine carotene xanthophylls, astaxanthin and fucoxanthin, possess potent anti-stress, anti-protein aggregation, and differentiation-inducing activities that warrant further mechanistic investigations for their recruitment in stress/disease prevention and therapeutics.

## 4. Materials and Methods

### 4.1. Cell Culture and Reagents

Rat glioma cells (C6) (obtained from the Cell Resource Center for Biomedical Research, Tohoku University, Sendai, Japan) were cultured in Dulbecco’s modified Eagle’s medium (Invitrogen)-supplemented with 5% fetal bovine serum and 1% penicillin/streptomycin in a humidified incubator (37 °C and 5% CO_2_). The marine carotenoids, astaxanthin (Supreme Health New Zealand Limited, Auckland, New Zealand; Batch # SB10012102013B; mol. weight 596.84 containing 5% free and 95% di/mono ester forms) and fucoxanthin (Wako, 063-06691; CAS number 3351-86-8; mol. weight 658.91, 95% purity by weight) were dissolved in DMSO to make 100 mM and 5 mM stocks, respectively, and stored at −20 °C. Sodium (meta)arsenite was dissolved in ultrapure water to make a 100 mM stock and stored at 4 °C. A UV chamber (FS-800, FUNA^®^-UV-linker) was used to induce UV-radiation stress. Antibodies against luciferase (Abcam, Cambridge, UK, ab16466), GFAP (Sigma-Aldrich, St. Louis, MO, USA, G9269), NF200 (Sigma-Aldrich, N4142), PSD95 (Santa Cruz, Dallas, TX, USA, sc-32290), MAP2 (Sigma-Aldrich, M3696), nestin (Santa Cruz, sc-23927), neuropsin (Santa Cruz, sc-134600), GAP43 (Santa Cruz, sc-33705), NCAM (Santa Cruz, sc-10735), survivin (Santa Cruz, sc-10811), vimentin (Santa Cruz, sc-6260), γH2AX (Cell Signaling, Danvers, MA, USA, 9718S), HP1γ (Merck Millipore, Burlington, MA, USA, 05-690), pChk1 (Cell Signaling, 2344S), pChk2 (Cell Signaling, 2197P), mortalin [64], p53 (Santa Cruz, SC-126), MRE11 (Novus Biologicals, Centennial, CO, USA, NB100-142), Rad50 (Cell Signaling, 3427S), NBS1 (Abcam, ab32074), pATR (Santa Cruz, sc-109912), PARP1 (Santa Cruz, sc-7150), 53BP1 (Abcam, ab21083), BRCA (Cell Signaling, 9010S), and β-actin (Abcam, ab49900) proteins were used for immunoblotting and immunostaining. Primers for MAP2 (F-CGAACTTTATATTTTACCACTTCCTTG/R-CCGTTCATCTGCCATTCTTC), nestin (F-GAATCAGATCGCTCAGATCC/R-GCACGACACCAGTAGAACTGG), GAP43 (F-ATGCTGTGCTGTATGAGAAGAACC/R-GGCAACGTGGAAAGCCGTTTCTTAAAG), and GAPDH (F-TGGAAATCCCATCACCATCT/R-TTCACACCCATGACGAACAT) were used for mRNA estimation in reverse transcriptase polymerase chain reaction.

### 4.2. Dose Titration

Cytotoxicity of the astaxanthin, fucoxanthin, sodium (meta)arsenite, and UV radiation in C6 cells were determined by MTT (3-(4,5-dimethylthiazol-2-yl)-2,5-diphenyltetrazolium bromide) assay. Two thousand cells per well were plated in a 96-well plate, allowed to settle overnight, and treated with varying doses of the compounds/stressors. The control (DMSO) or treated cells were incubated for 48 h followed by addition of 10 µL of phosphate buffered saline (PBS) containing 5 mg/mL MTT (M6494, Life Technologies, Carlsbad, CA, USA), and further incubated for 4 h. Culture medium containing MTT was aspirated and replaced with DMSO. The plates were placed on a shaker for 5 min followed by measurement of optical density at 570 nm using Tecan infinite M200^®^ Pro microplate reader (Tecan Group Ltd., Mannedorf, Switzerland). Cell viability was calculated as a percentage against the control to identify their IC_01_ value using Microsoft™ Office^©^ 2016. Statistical significance was calculated by an unpaired *t*-test of GraphPad^®^ software (2018) using mean, SD, and N from three independent experiments, and shown as * *p* < 0.05, ** *p* < 0.01, *** *p* < 0.001, ns = not significant.

### 4.3. QCV Assay

Five hundred cells per well were plated in a 6-well plate and allowed to settle overnight, followed by treatment with varying doses of the marine carotenoids. The control or drug-treated cells were incubated at 37 °C and 5% CO_2_. The drug-supplemented medium was replaced every alternate day. After 8 days, cells were fixed, stained, and de-stained into the solution, which was quantified by the help of a spectrophotometer as described previously [2]. Statistical significance was calculated by an unpaired *t*-test of GraphPad^®^ software (2018) using mean, SD, and N from three independent experiments, and shown as * *p* < 0.05, ** *p* < 0.01, *** *p* < 0.001, ns = not significant.

### 4.4. Cell Proliferation

In order to estimate the proliferative rate of cells, five thousand cells were plated in 6-well plates and allowed to settle overnight, followed by culture either in control or drug-supplemented medium. Cells were fixed in each variant of treatment and counted every day for 8 days. A proliferation histogram was plotted considering control on day 1 as 100 percent using Microsoft™ Office^©^ 2016. Statistical significance was calculated by an unpaired *t*-test of GraphPad^®^ software (2018) using mean, SD, and N from three independent experiments, and shown as *** *p* < 0.001, ns = not significant.

### 4.5. Cell Differentiation and Crystal Violet Staining

Two thousand cells per well were plated in a 6-well plate, allowed to settle overnight, and treated with varying doses of the compounds/stressors. The drug-supplemented medium was replaced every alternate day for 28 days. Cells were then visualized under a phase contrast microscope and recorded at X400 magnification. Differentiated cells were counted manually as random 100 selections by ImageJ software 1.52a (NIH), and tabulated and averaged using Microsoft™ Office^©^ 2016. Statistical significance was calculated by an unpaired *t*-test of GraphPad^®^ software (2018) using mean, SD, and N from three independent experiments, and shown as *** *p* < 0.001, ns = not significant.

### 4.6. Stress Protection

Two thousand cells per well were plated in two 96-well plates and allowed to settle overnight. Cells in the first plate were pretreated with varying doses of the compounds/stressors. The control (DMSO) or treated cells were incubated for 24 h, following which the cells in both the plates were stressed with UV stress (a well-established model of DNA and oxidative damage) at the rate of 5 mJ/cm^2^ and recovered in either control or drug-supplemented medium for 48 h (plate 1 = pretreatment model, plate 2 = recovery model), followed by addition of 10 µL of phosphate buffered saline (PBS) containing 5 mg/mL MTT (M6494, Life Technologies), and further incubated for 4 h. Culture medium containing MTT was aspirated and replaced with DMSO. The plates were placed on a shaker for 5 min followed by measurement of optical density at 570 nm using Tecan infinite M200^®^ Pro microplate reader (Tecan Group Ltd., Mannedorf, Switzerland). Cell viability was calculated as a percentage against the control using Microsoft™ Office^©^ 2016. The experiment was performed three times, and the histograms bearing cumulated data were plotted. Statistical significance was calculated by an unpaired *t*-test of GraphPad^®^ software (2018) using mean, SD, and N from four independent experiments, and shown as * *p* < 0.05, ** *p* < 0.01, *** *p* < 0.001, ns = not significant.

### 4.7. Comet Assay

One hundred thousand cells per well were plated in a 6-well plate, allowed to settle overnight, and pretreated with varying doses of the compounds. After 24 h of pretreatment, the cells were stressed with UV radiation at the rate of 3 mJ/cm^2^, with 50 mJ/cm^2^ as the positive control, and recovered in the drug-supplemented medium. The protective effect of the marine carotenoids against UV-mediated DNA damage was evaluated using a single cell gel neutral comet assay (Trevigen’s Comet Assay^®^) electrophoresis system following the manufacturer’s protocol. Comet tail length was calculated by ImageJ software (NIH) and tabulated as a percentage against the control using Microsoft™ Office^©^ 2016. Statistical significance was calculated by an unpaired *t*-test of GraphPad^®^ software (2018) using mean, SD, and N from three independent experiments, and shown as *** *p* < 0.001.

### 4.8. Generation of Stable GFP-Expressing Cells

Five thousand cells per well were plated in a 12-well plate and allowed to settle overnight. Cells were transfected with 100 ng of plasmid-expressing GFP from a constitutive (β-actin) promoter using Lipofectamine™ 2000 transfection reagent (Thermo Fisher Scientific, Waltham, MA, USA, 11668027) in Opti-MEM™ reduced serum medium (Gibco™, 10149832). Forty-eight hours later, the transfection efficiency was determined by direct observation under the microscope. The cells with more than 50% transfection were processed through selection with Dulbecco’s Modified Eagle’s medium (DMEM) supplemented with 0.5 mg/mL hygromycin B (Clontech, Mountain View, CA, USA, 8057-1) for 96 h. Colonies of GFP-expressing C6 cells were then identified and marked under the microscope, transferred onto a fresh culture dish manually, and allowed to grow under favorable conditions to form a stable GFP-expressing C6 cell line.

### 4.9. Protein Aggregation and Deaggregation

Fifty thousand GFP-expressing C6 cells per well were plated in a 6-well plate, allowed to settle overnight, and pretreated with varying doses of the compounds. After 24 h, the cells were stressed with sodium (meta)arsenite 20 µM for another 24 h, following which they were washed thoroughly with PBS thrice and recovered in the drug-supplemented medium for 48 h. Cells were then visualized under a fluorescent microscope and recorded at 400× magnification. Aggregates were quantified using ImageJ software (NIH) and plotted as a percentage using Microsoft™ Office^©^ 2016. Statistical significance was calculated by an unpaired *t*-test of GraphPad^®^ software (2018) in stressed-treated samples using mean, SD, and N from four independent experiments against the positive control, and shown as ** *p* < 0.01, *** *p* < 0.001.

### 4.10. Heat-Shock Aggregation of Luciferase

One hundred thousand cells per well were plated in a 6-well plate in two identical sets and allowed to settle overnight. The cells were transfected with pGL4-p53-3′ UTR expressing luciferase from a constitutive promoter as described earlier. After 48 h, cells were heat shocked at 42 °C and 5% CO_2_ for 2 h, followed by recovery at 37 °C either in the control or drug-supplemented medium for the next 48 h. The first set of cells were taken for immunocytochemistry for luciferase protein as described later in Section 4.12, and the second was lysed using passive lysis buffer for luciferase expression estimation using the luciferase assay system (Promega, Madison, WI, USA, E1501) following the manufacturer’s protocol. Protein expression was quantified by ImageJ software (NIH) and tabulated with reporter luciferase luminescence values as a percentage against the control using Microsoft™ Office^©^ 2016. Statistical significance was calculated by an unpaired *t*-test of GraphPad^®^ software (2018) using mean, SD, and N from three independent experiments, and shown as *** *p* < 0.001.

### 4.11. Immunoblotting

Two hundred thousand cells per well were plated in a 6-well plate and allowed to settle overnight, followed by the treatment with varying doses of the compounds/stressors. Control and treated cells were harvested and washed with PBS (X2), followed by lysis in RIPA buffer (89900, Thermo Fisher Scientific) containing complete protease inhibitor cocktail (4693159001, Roche Applied Science, Penzberg, Bavaria, Germany) on ice for 45 min. Lysates were separated on an SDS-polyacrylamide gel using Mini-Protean^®^ Tetra cell equipment (Bio-Rad, Hercules, CA, USA), and subjected to western blotting using protein-specific antibodies as indicated and horseradish peroxidase-conjugated secondary HRP antibody (31430 or 31460, Thermo Fisher Scientific). Blots were developed using chemiluminescence solution (GE Healthcare, Buckinghamshire, UK) and visualized using a Lumino Image Analyzer (LAS 3000-mini; Fuji Film, Tokyo, Japan). Band intensity was quantified using ImageJ software (NIH) and plotted as a percentage using Microsoft™ Office^©^ 2016. Statistical significance was calculated by an unpaired *t*-test of GraphPad^®^ software (2018) using mean, SD, and N from four independent experiments, and shown as * *p* < 0.05, ** *p* < 0.01, *** *p* < 0.001, ns = not significant.

### 4.12. Immunostaining

Twenty-five thousand cells per well were plated on glass coverslips placed in 12-well cell culture plates. After 48 h of treatment with drugs/stressors, control or treated cells were fixed in methanol:acetone (1:1). Cells were permeabilized with Tween-20 in phosphate buffered saline (PBST), washed with phosphate buffered saline (PBS), and blocked with 2% bovine serum albumin protein dissolved in PBST. Fixed cells were incubated with primary antibodies (as indicated) overnight, washed with PBS-PBST-PBS (5 min each), incubated with either Alexa-Fluor 488 goat anti-mouse IgG (Life Technologies, A11029) or Alexa-Fluor 594 goat anti-rabbit IgG (Life Technologies, A11037), depending upon the source of the primary antibodies, for 2 h, washed with PBS-PBST-PBS (5 min each), incubated with Hoechst 33342 stain (Invitrogen Molecular Probes, Carlsbad, CA, USA, H3570) for 10 min, washed with PBST-PBS-ultrapure water (5 min each), and mounted on glass slides. The cells were then visualized for immunofluorescence under a microscope at 400× magnification. Protein expression was quantified using ImageJ software (NIH) and plotted as a percentage using Microsoft™ Office^©^ 2016. Statistical significance was calculated by an unpaired *t* test of GraphPad^®^ software (2018) using mean, SD, and N from three independent experiments, and shown as * *p* < 0.05, ** *p* < 0.01, *** *p* < 0.001, ns = not significant.

### 4.13. Reverse Transcriptase Polymerase Chain Reaction

Two thousand cells per well were plated in a 6-well plate, allowed to settle overnight, and treated with varying doses of the compounds/stressors. The drug-supplemented medium was replaced every alternate day for 25 to 35 days until the appearance of differentiated morphology in the cells (described in earlier sections). Cells were harvested from the Petri dishes and lysed with Trizol (Ambion^®^, Foster City, CA, USA, 15596018) for 5 min at room temperature, segregated in chloroform (Wako, Tokyo, Japan, 038-02606) for 5 min at room temperature, centrifuged at 12,000 rpm for 15 min and supernatant separated, washed in isopropanol (Wako, 166-04836) for 10 min at room temperature, centrifuged at 12,000 rpm for 15 min and pellet washed in 70% ice-cold ethanol and centrifuged at 8000 rpm for 5 min twice, followed by air-drying and resuspension in nuclease-free water to extract pure RNA. The concentration and quality of RNA were evaluated through a spectrophotometer (ND-1000, Nanodrops^®^, Wilmington, NC, USA). cDNA was prepared using a reverse transcription kit (Qiagen, Hilden, Germany, 205313) following the manufacturer’s instructions. The master mix for amplification was prepared by mixing 1 µL cDNA with 0.1 µL Ex Taq (Takara, Kusatsu, Shiga, Japan, RR001), 2 µL 10× TAQ buffer, 2 µL dNTP, 1 µL each of forward and reverse primers (indicated earlier) in 12.9 µL nuclease free water and amplified using ‘denaturation–95 °C, 10 min → amplification–95 °C, 45 s–60 °C, 1 min–72 °C, 45 s (35~48 cycles) → annealing–72 °C, 10 min → 4 °C’ protocol. The amplified products were separated on a 1% agarose gel containing 0.0625 µg/mL ethidium bromide (Invitrogen^®^, 15585-011), and acquired using a Lumino Image Analyzer (LAS3000-mini; Fuji Film, Tokyo, Japan) equipped with a CCD (Charge-coupled device) camera. Band intensity was quantified using ImageJ software (NIH) and plotted as a percentage using Microsoft™ Office^©^ 2016. Statistical significance was calculated by an unpaired *t*-test of GraphPad^®^ software (2018) using mean, SD, and N from four independent experiments, and shown as * *p* < 0.05, *** *p* < 0.001.

### 4.14. Statistical Analysis

All the quantifications were performed using ImageJ software (NIH), calculations were done using Microsoft™ Office^©^ 2016, and plotted as percentages. Statistical significance was calculated by an unpaired *t*-test of GraphPad^®^ software (2018) using mean, SD, and N from three independent experiments, and shown as * *p* < 0.05, ** *p* < 0.01, *** *p* < 0.001, ns = not significant.

## Supplementary Materials

The following are available online at https://www.mdpi.com/1660-3397/17/3/189/s1, Figure S1. 2-D and 3-D chemical structures of the two selected marine carotenoids, Astaxanthin and Fucoxanthin; Figure S2. Schematic presentation of the effect of Astaxanthin and Fucoxanthin on C6 cell differentiation determining proteins.
