# Supplementary material for: Rat Glioma Cell-Based Functional Characterization of Anti-Stress and Protein Deaggregation Activities in the Marine Carotenoids, Astaxanthin and Fucoxanthin
Source: Mar Drugs. 2019 Mar 23;17(3):189. doi: 10.3390/md17030189 (PMC6470788; doi:10.3390/md17030189)
Supplement: Supplementary file 1 [file marinedrugs-17-00189-s001.pdf]

SUPPLEMENTARY INFORMATION

Cell-based Functional Characterization of Anti-stress and Protein deaggregation Activities in the Marine Carotenoids, Astaxanthin and Fucoxanthin

Sajal Afzal[1,2], Sukant Garg[1], Yoshiyuki Ishida[3], Keiji Terao[3], Sunil C. Kaul[1] and Renu Wadhwa[1,2*]

[1]DAILAB, DBT-AIST International Center for Translational and Environmental Research (DAICENTER), National Institute of Advanced Industrial Science & Technology (AIST), Tsukuba 305-8565, Japan, [2]School of Integrative and Global Majors, University of Tsukuba, Tsukuba 305-8577, Japan and [3]CycloChem Co., Ltd., 7-4-5 Minatojima-minamimachi, Chuo-ku, Kobe - 650 0047, Japan

sajal.afzal@aist.go.jp (S.A)

sukantgarg@gmail.com (S.G)

yoshiyuki.ishida@cyclochem.com (Y.I)

keiji.terao@cyclochem.com (K.T.)

s-kaul@aist.go.jp (S.C.K)

*Correspondence: renu-wadhwa@aist.go.jp (R.W.), Tel: +81-29-861-9464

Supplementary Figure 1

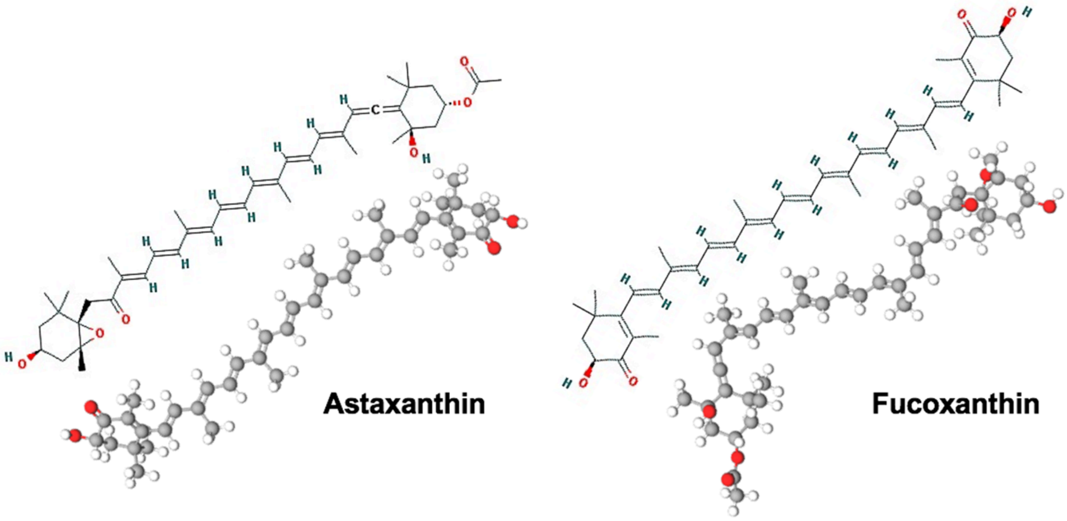

Supplementary Fig. 1. 2-D and 3-D chemical structures of the two selected marine carotenoids, Astaxanthin and Fucoxanthin.

Supplementary Figure 2

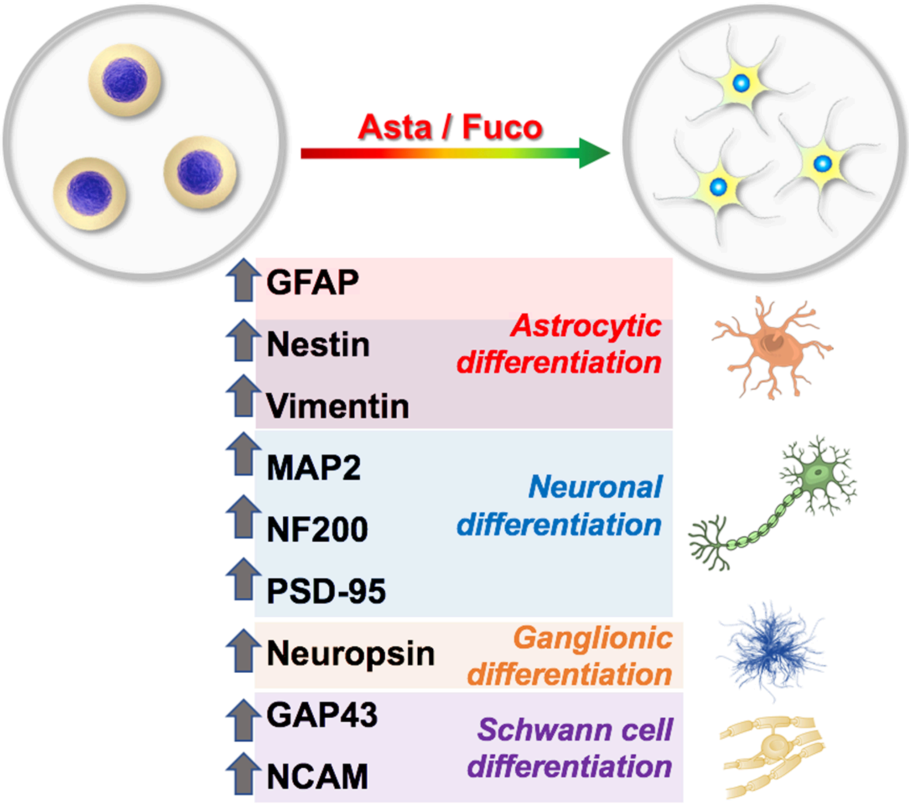

Supplementary Fig. 2. Flowchart depicted neural maturation stages and hallmark associated protein markers.
